# Effects of Tobacco-Smoking on Children

**Published:** 1870-07

**Authors:** 


Effects of Tobacco-Smoking on Children.—It seems that the habit of smoking has taken possession of the boys in France to such an extent as to elicit serious inquiry as to its results. An able writer describes his experience on the subject, and comes to the following conclusions:—1. The pernicious effects on boys are incontestable. 2. They consist of pallor, chloro-anæmia, palpitations of the heart, diminution of the normal number of red globules, and impaired digestion. 3. The ordinary treatment for anemia, etc, is ineffectual so long as the habit of smoking is persisted in. 4. Boys who are addicted to smoking, exhibit a want of intelligence, and have a liking more or less decided for strong drinks. 5. Those who abandon the practice before any serious organic lesions are produced recover their health perfectly.

